# Integrated analysis of proteomics and metabolomics in girls with central precocious puberty

**DOI:** 10.3389/fendo.2022.951552

**Published:** 2022-07-28

**Authors:** Mei Li, Dan Lan, Yanfei Chen

**Affiliations:** Department of Pediatrics, The First Affiliated Hospital of Guangxi Medical University, Nanning, China

**Keywords:** central precocious puberty (CPP), proteomics, metabolomics, lipid pathway, biomarker

## Abstract

**Background:**

Central precocious puberty (CPP) is a multifactorial and complex condition. Traditional studies focusing on a single indicator cannot always elucidate this panoramic condition but these may be revealed by using omics techniques.

**Objective:**

Proteomics and metabolomics analysis of girls with CPP were compared to normal controls and the potential biomarkers and pathways involved were explored.

**Methods:**

Serum proteins and metabolites from normal girls and those with CPP were compared by LC-MS/MS. Multivariate and univariate statistical analysis were used to identify the differentially expressed proteins (DEPs) and differentially expressed metabolites (DEMs). Functional annotation and pathway enrichment analysis were performed by using GO and KEGG databases, and candidate markers were screened. Finally, bioinformatic analysis was used to integrate the results of proteomics and metabolomics to find the key differential proteins, metabolites and potential biomarkers of CPP.

**Results:**

134 DEPs were identified in girls with CPP with 71 up- and 63 down-regulated, respectively. Up-regulated proteins were enriched mainly in the extracellular matrix, cell adhesion and cellular protein metabolic processes, platelet degranulation and skeletal system development. The down-regulated proteins were mainly enriched in the immune response. Candidate proteins including MMP9, TIMP1, SPP1, CDC42, POSTN, COL1A1, COL6A1, COL2A1 and BMP1, were found that may be related to pubertal development. 103 DEMs were identified, including 42 up-regulated and 61 down-regulated metabolites which were mainly enriched in lipid and taurine metabolic pathways. KGML network analysis showed that phosphocholine (16:1(9Z)/16:1(9Z)) was involved in arachidonic acid, glycerophospholipid, linoleic acid and α-linolenic acid metabolism and it may be used as a biomarker of CPP.

**Conclusions:**

Our study is the first to integrate proteomics and metabolomics to analyze the serum of girls with CPP and we found some key differential proteins and metabolites as well as a potential biomarker for this condition. Lipid metabolism pathways are involved and these may provide a key direction to further explore the molecular mechanisms and pathogenesis of CPP.

## Introduction

Sexual development in humans is a continuous process governed by certain rules. Central precocious puberty (CPP) in girls is caused by the early initiation of the hypothalamic-pituitary-gonadal axis (HPGA), which is characterized by rapid development of internal and external reproductive organs and secondary sexual characteristics before the age of 8, and the sequence of sexual development is basically the same as that of normal girls (1). Based on epidemiologic data, there has been a worldwide trend towards earlier onset of puberty and there is an increased incidence of precocious puberty (2–4). Interestingly, the incidence of precocious puberty has also increased during the lockdown due to the COVID-19 pandemic (5, 6). Apart from secondary CPP, the etiology and pathogenesis of this idiopathic condition are still unclear.

It is now widely accepted that the timing of puberty in humans is determined by complex interactions, including genetics, epigenetics, environmental, nutritional and the gut microbiome (7). The monogenic etiologies of CPP puberty that have been described so far include activating mutations in the KISS1/KISS1R system and inactivating mutations in the imprinted genes, MKRN3 and DLK1 (8). Although mutations in KISS1 and KISS1R had been previously identified (9, 10), no other CPP cases have been reported since 2010, suggesting these remain rare monogenic causes of this condition (11). CPP has a genetic predisposition, and mutations in MKRN3 are common monogenic causes of familial CPP (12). Inactivating of mutations in DLK1 are also associated with familial CPP (13), but these are not common monogenic causes (11). In addition to its monogenic etiology, obesity (14) and environmental endocrine disrupting chemicals (EDCs) (15) are currently considered to be the main causes of CPP. EDCs could regulate the activation of either gonadotropin-releasing hormone (GnRH) neurons or gonadal steroidogenesis in order to initiate puberty through epigenetic mechanisms (16). Recently, Heras et al. (17) revealed that linking kisspeptin, hypothalamic paraventricular nucleus (PVN) ceramide synthesis and sympathetic innervation in the rat ovary were key to obesity-induced pubertal precocity. Although more and more studies have attempted to explain the occurrence of CPP, the regulatory network involved in puberty remains a mystery.

In recent years, with the rapid development of post-genomic techniques, the integration of multi-omics data has been widely used to understand complex diseases (18). Proteomics is a science that studies the protein composition and changes that occur in cells, tissues and whole organisms, and this was first proposed by Wilkins in 1996 (19). Metabolomics is an emerging field that provides a comprehensive coverage of biological processes and metabolic pathways by providing a large number of metabolite analyses (20). Therefore, an integrated approach that combines proteomics and metabolomics can be a potentially very powerful tool that can provide an advisable strategy for exploring biomarkers and the molecular mechanisms of diseases. CPP is a disease with complex etiology and pathogenesis, and a reliance on traditional research methods cannot meet the all needs of research into this condition. In our study, we performed an integrated proteomics and metabolomics analysis in order to explore the pathways that were altered in girls with CPP and to reveal potential biomarkers that can aid in its diagnosis.

## Materials and methods

### Ethics

This study conformed to the Declaration of Helsinki and was approved by the Scientific Ethics Committee of The First Affiliated Hospital of Guangxi Medical University in Nanning, China (2022 (KY-E-025)). Informed consent was obtained from all participants or their legal guardians.

### Patients

This study was conducted in the First Affiliated Hospital of Guangxi Medical University from May to December 2021. 10 girls with CPP and 10 age-matched prepubertal healthy girls were selected for data independent acquisition (DIA) quantitative proteomics. In addition, 31 girls with CPP and 19 age-matched prepubertal healthy girls were selected for untargeted metabolomics. Among them, proteomic and metabolomic tests were performed on 10 girls with CPP and 10 age-matched controls, and all the data were analyzed jointly. Anthropometric, sexual development and gonadal hormone assessments were collected for all subjects (**Table 1**). With respect to the criteria for inclusion of the CPP cases, this was referred to the Consensus on diagnosis and treatment of CPP, (2015) (1) and these included: 1) Breast development before 8 years of age; 2) Linear growth acceleration where the annual growth rate was higher than in normal girls; 3) Progressive bone age was more than one year of the chronological age; 4) The pelvic ultrasound of the uterus was 3.4 ~ 4.0cm in length with an ovarian volume of 1 ~ 3mL and the presence of multiple follicles ≥4mm in diameter; 5) The peak level of luteinizing hormone (LH peak) ≥ 5 IU/L and the LH peak/follicle-stimulating hormone (FSH) peak ≥0.6 after GnRH simulation. Girls with CPP but with central nervous system (CNS) abnormalities and other secondary diseases, such as congenital adrenal hyperplasia, McCune-Albright syndrome, and primary hypothyroidism, were excluded from the study.

**Table 1:** Anthropometric and hormone characteristics of the research subjects in this study.

Items		Proteomics						Metabolomics					
		CPP (n=10)			Control (n=10)		P	CPP (n=31)			Control (n=19)		P
Age (y)		8.57 ± 0.77			8.46 ± 0.79		0.756	8.17 ± 0.91			7.88 ± 0.93		0.274
Tanner’s stage	Breast	II	III	IV	I	II		II	III	IV	I	II	
		4 (40%)	5 (50%)	1 (10%)	9 (90%)	1 (10%)	–	20 (64.5%)	8 (25.8%)	3 (9.7%)	17 (89.5%)	2 (10.5%)	–
	Pubic hair	I	II		I		–	I	II	III	I		–
		7 (70%)	3 (30%)		10 (100%)		–	26 (83.9%)	4 (12.9%)	1 (3.2%)	19 (100%)		–
Height (cm)		132.15 ± 7.45			126.80 ± 5.87		0.091	132.87 ± 6.93			124.66 ± 5.36		<0.001
Weight (kg)		30.65 ± 5.89			24.69 ± 2.19		0.012	28.36 ± 5.18			23.69 ± 3.34		0.001
Height z score		0.04			0.03		–	0.05			0.03		–
BMI (kg/m2)		17.38 ± 1.72			15.37 ± 1.09		0.007	16.15 ± 1.56			15.22 ± 1.65		0.054
BMI z score		0.10			0.08		–	0.09			0.12		–
B-LH (IU/L)		2.85 (1.25, 6.20)			0.06(0.03, 0.12)		<0.001	1.87 (0.70, 4.09)			0.06 (0.03, 0.08)		<0.001
P-LH(IU/L)		46.26 (14.73, 78.32)			–		–	20.27 (11.52, 59.97)			–		–
B-FSH (IU/L)		4.46 (3.55, 5.27)			1.23 (1.05, 1.84)		<0.001	4.65 (3.15, 5.96)			1.32 (1.10, 1.77)		<0.001
P-FSH(IU/L)		17.38 (13.66, 27.46)			–		–	17.21 (14.16, 24.12)			–		–
E2 (pmol/L)		40.17 (25.01, 61.37)			26.87 (19.39, 32.87)		0.105	37.18 (29.23, 55.49)			25.01 (19.49, 28.63)		0.002

Values are expressed as the means ± standard deviations for normally distributed variables or expressed as medians (interquartile ranges) for non-normally distributed variables. B- LH, basal luteinizing hormone; B-FSH, basal follicle stimulating hormone; E2, estradiol; P-LH, peak luteinizing hormone; P-FSH, peak follicle stimulating hormone.

### Serum samples collection

Venous blood samples were collected early in the morning after fasting for at least 8 hours and placed in a dry blood collection tube. The samples were then centrifuged at 4244g at 4°C for 10 minutes. Serum samples were collected and stored at -80°C. Serum samples of girls with CPP were collected before initiation of GnRHa treatment.

### Proteomics

In our study, serum samples from 20 subjects (10 girls with CPP and 10 controls) were analyzed by DIA quantitative proteomics analysis. The total protein in the sample was extracted and some were used to determine the protein concentration as well as for SDS-PAGE, and the rest was subjected to trypsin digestion. After desalting, LC-MS/MS was used to identify the peptides in the samples. Firstly, a protein spectrum library was established by using the traditional data dependent acquisition (DDA) method, and then the mass spectrometry data of each sample were obtained by using DIA technology. Spectronaut pulsar software was used to search all the raw data thoroughly against the known protein databases (UniProtKB). A database search was performed specifically for trypsin digested samples. Alkylation of cysteine was considered as a fixed modification during the database search. The proteins, peptides and peptide-to-spectrum matched false discovery rate (FDR) were all set to 0.01. For DIA data, the quantification of FDR was set to 0.05 and the quantity MS-level was set at MS2. The differentially expressed protein (DEPs) were identified using the following criteria: 1) Fold change>1.2 or <0.83; 2) p value<0.05 (t-test between the two groups). DEPs were submitted to the DAVID website (https://david.ncifcrf.gov/home.jsp) for functional annotation. Terms of biological processes, cellular component and molecular function were analyzed according to the Gene Ontology (GO) database (http://amigo.geneontology.org) and functional pathway analysis was based on the Kyoto Encyclopedia of Genes and Genomes (KEGG) (21–23) database (http://www.kegg.jp/). Hypergeometric distribution test was used to determine the significance of DEP-enrichment in each GO term or KEGG pathway. A pathway enrichment test p value of less than 0.05, and proteins counts of more than 5 were set as the screening criteria for results.

### Metabolomics

Serum samples from 50 subjects (31 girls with CPP and 19 controls) were analyzed by untargeted metabolomics. A Dionex U3000 UHPLC ultra high performance liquid series QE PLUS high resolution mass spectrometer, composed of liquid-mass coupling system was used for analysis of samples. Progenesis QI software (Waters Corporation, Milford, USA) was used to analyze the LC-MS raw data. Three-dimensional datasets such as m/z, peak RT and peak intensity were formed into an Excel file, and RT–m/z pairs were used as the identifiers of each ion. The resulting matrix was further reduced by removing any peaks with missing values (ion intensity=0) in more than 50% of the samples. An internal standard was used for data quality control (QC; reproducibility). QC samples were prepared by mixing aliquots of all the samples into a pooled sample. The metabolites were identified by using Progenesis QI Data Processing Software, based on publically available databases such as http://www.hmdb.ca/ ; http://www.lipidmaps.org/ as well as self-built databases. The positive and negative data were combined to obtain a combined dataset which was imported into the R ropls package. Principle component analysis (PCA) and orthogonal partial least-squares-discriminant analysis (OPLS-DA) were used to observe the metabolic alterations between the two groups, after mean centering (Ctr) and Pareto variance (ParV) scaling, respectively. Variable importance in the projection (VIP) ranked the overall contribution of each variable to the OPLS-DA model. To prevent overfitting of the model, 7-round cross-validation and 200 response permutation testing (RPT) were used to evaluate the quality of the model. Metabolites with VIP values larger than 1.0 and p values less than 0.05 were considered to be DEMs between the groups.

### Statistical analysis

Statistical analysis was performed by using SPSS Statistics Version 23.0 software (IBM SPSS, Armonk, NY, USA). Data was tested for normality of distribution by using the Shapiro–Wilk test. Clinical variables were compared between groups using t-test for normally distributed data, and the Mann-Whitney U test was used for non-normally distributed data. R software (version 3.6.2) was used for statistical analyses. The ropls package analysis software was used for multivariate statistical analysis (PCA, OPLS-DA and OPLS). Analysis packages included pheatmap, ggplot2 and ggrepel and correlation analysis was performed using corrplot. Univariate statistical correlation was performed by using a basic software package. Statistical significance was defined as a p value equal to or less than 0.05.

## Results

### Proteomic analysis

**Figure 1:**
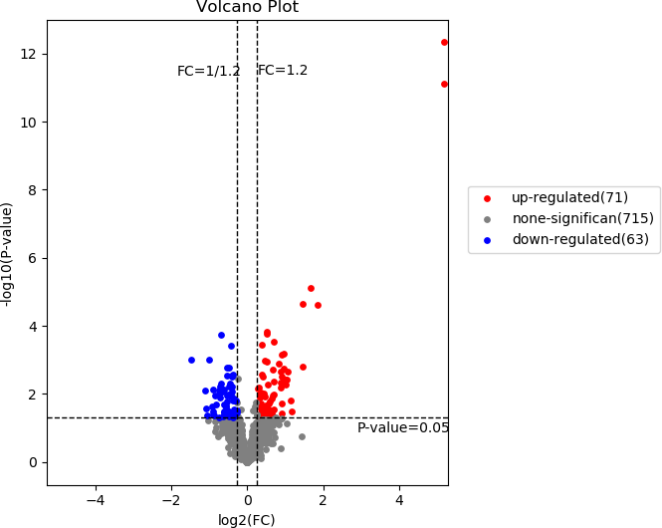
A volcano plot of the differentially expressed proteins. The red and blue dots represent significantly up-regulated and down-regulated DEPs, respectively. The horizontal dotted line represents p value <0.05, two vertical dashed lines indicate Fold change=1.2 and Fold change=0.83.

Our study identified 10009 peptides and quantified 1002 proteins. Of these, 134 quantified proteins were identified as DEPs, of which 71 were up-regulated and 63 were down-regulated in the CPP group when compared to the normal group (>1.2-fold or <0.83-fold). The 134 DEPs were visualized by using a volcano plot as shown in **Figure 1**.

**Figure 2:**
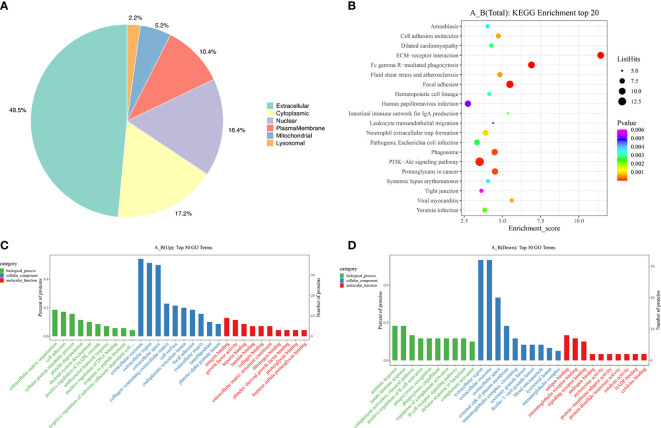
Subcellular localization and functional enrichment analysis of differentially expressed proteins. **(A)** Subcellular localization of the differentially expressed proteins. **(C)** GO terms of the differentially expressed proteins in the up-regulated group. **(D)** GO terms of the differentially expressed proteins in the down-regulated group. **(B)** The top 20 bubbles of KEGG enrichment in the differentially expressed proteins.

After the DEPs were obtained, GO/KEGG enrichment analysis was performed to describe their functions. The subcellular localization of 134 DEPs were analyzed. The top three subcellular localization were extracellular (48.5%), cytoplasmic (17.2%) and nuclear (16.4%) as shown in **Figure 2A**. Then, all the DEPs were classified into biological process (BP), cellular component (CC) and molecular function (MF) based on three probable functions. For the up-regulated proteins, the top three of BP were extracellular matrix organization, cell adhesion and cellular protein metabolic process (**Figure 2C**, green label). The top three of CC were extracellular exosome, extracellular region and extracellular space (**Figure 2C**, blue label). This is consistent with the subcellular localization analysis which showed that 48.5% of the DEPs were located extracellularly (**Figure 2A**). The top ten of MF were mainly related to binding functions (**Figure 2C**, red label). For the down-regulated proteins, the top three of BP were immune response, innate immune response and complement activation *via* the classical pathway (**Figure 2D**, green label). The top three of CC (**Figure 2D**, blue label) and the top ten of MF (**Figure 2D**, red label) were similar to those of the up-regulated proteins. The top 20 of the KEGG enrichment pathways based on the DEPs between girls with CPP and the normal group are shown in **Figure 2B** (see the supplement datasets for all the significant terms). ECM-receptor interaction, Fc gamma R-mediated phagocytosis, focal adhesion, PI3K-Akt signaling pathway, phagosome and proteoglycans in cancer were the major significant KEGG enrichment pathways obtained from the analysis.

**Figure 3:**
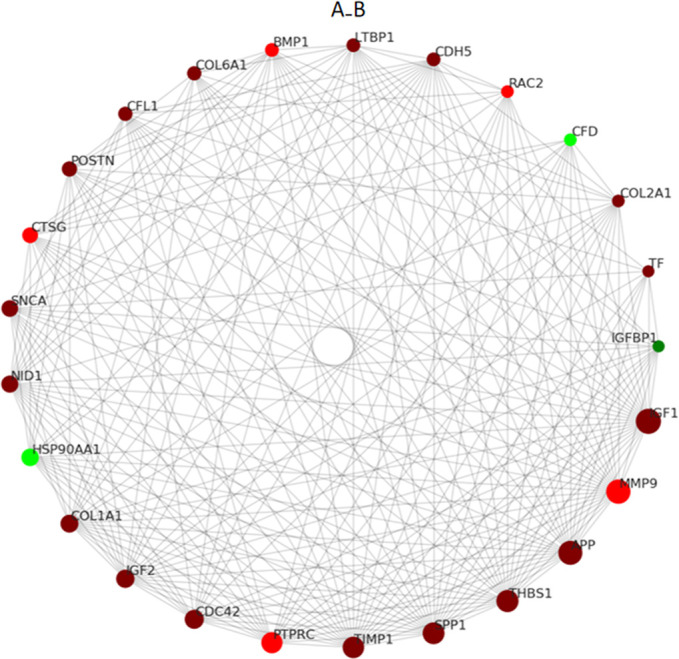
PPI network of the differentially expressed proteins in CPP. The circles in the figure indicate differentially expressed proteins, red circles represent up-regulated proteins and green circles represents down-regulated proteins. The size of the circle represents the degree of connection, and the larger the circle, the more connected it is.

In order to obtain the interaction relationships of DEPs, the top 25 proteins with connectivity were selected and a protein-protein interaction (PPI) network diagram was drawn by searching the STRING database (https://string-db.org). The first 25 nodes with connectivity were visualized by using the Python package “network” and these are shown as protein IDs (**Figure 3**). Among these, only 3 proteins were down-regulated, and the remaining 22 proteins were up-regulated. Then, the network topology was analyzed, and the 25 proteins were displayed according to their degree of involvement as shown in **Table 2**.

**Table 2:** A list of topological information of the network nodes involved.

Accession	Gene Name	FC	Degree
P05019	IGF1	1.567642216	62
P14780	MMP9	2.071147156	59
P05067	APP	1.402941913	58
P07996	THBS1	1.6143275	52
P10451	SPP1	1.388791725	51
P01033	TIMP1	1.548383073	50
P08575	PTPRC	1.876677053	49
P60953	CDC42	1.602653542	43
P01344	IGF2	1.343936209	41
P02452	COL1A1	1.623211919	40
P07900	HSP90AA1	0.729889712	39
P14543	NID1	1.255191999	38
P37840	SNCA	1.579364545	37
P08311	CTSG	2.095382839	35
Q15063	POSTN	1.386956741	34
P23528	CFL1	1.480656624	32
P12109	COL6A1	1.228885387	32
P13497	BMP1	2.225164023	31
Q14766	LTBP1	1.442962676	31
P33151	CDH5	1.228331459	31
P15153	RAC2	1.862280196	29
P02458	COL2A1	1.283357367	29
P00746	CFD	0.754562337	29
P02787	TF	1.239288	28
P08833	IGFBP1	0.531224263	28

### Metabolomic analysis

**Figure 4:**
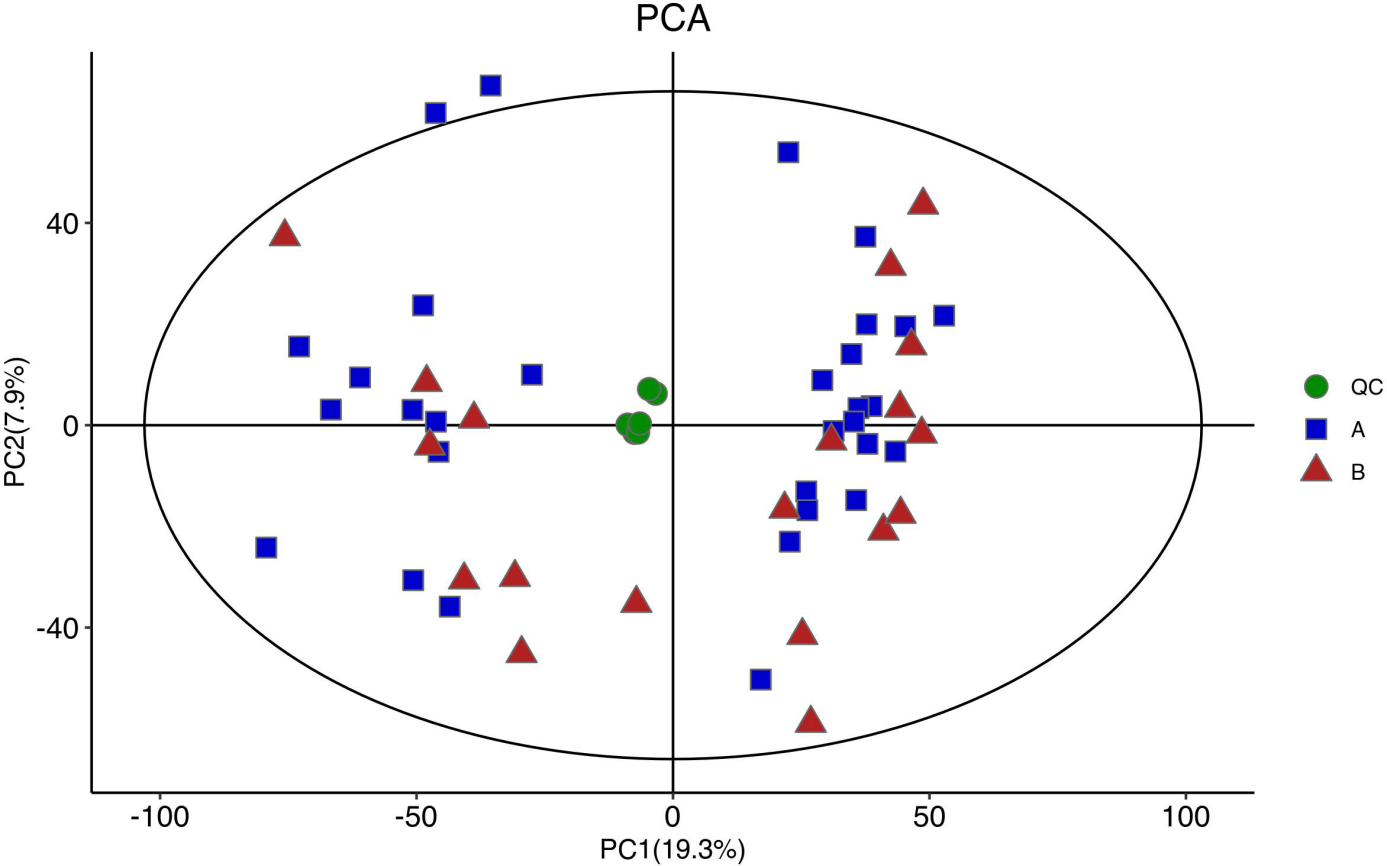
A PCA diagram of the samples used for analysis (including QC). QC samples were closely clustered in the middle of all samples, and no outlier samples were found, indicating good stability of instrumental analysis system and stable and reliable experimental data.

**Figure 5:**
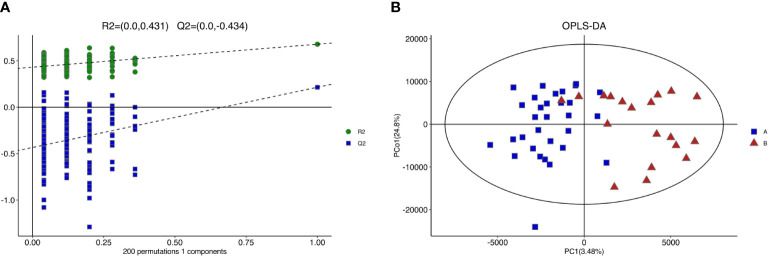
Results of replacement test and OPLS-DA score of girls with CPP vs the control group. **(A)** The closer R2Y was to 1, the more stable and effective the model was. Q2<0 indicated that the model was reliable and effective without over-fitting. **(B)** There was significant difference in OPLS-DA score between the two groups.

**Figure 6:**
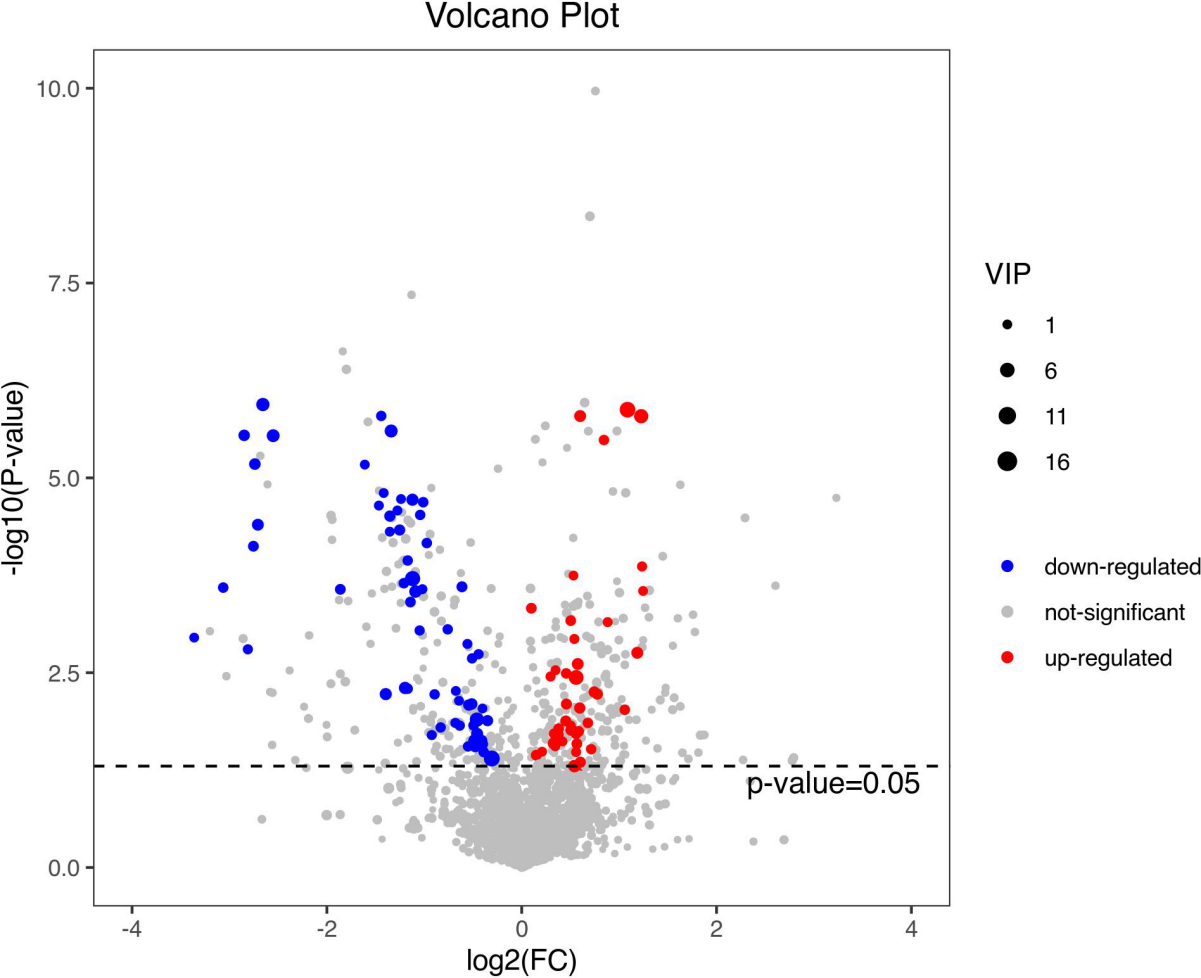
A volcano plot of the differential metabolites. The volcano map can be used to visualize p value, VIP value and FC value, which is beneficial to screen differential metabolites. The red and blue dots represent significantly up-regulated and down-regulated DEMs, respectively.

**Figure 7:**
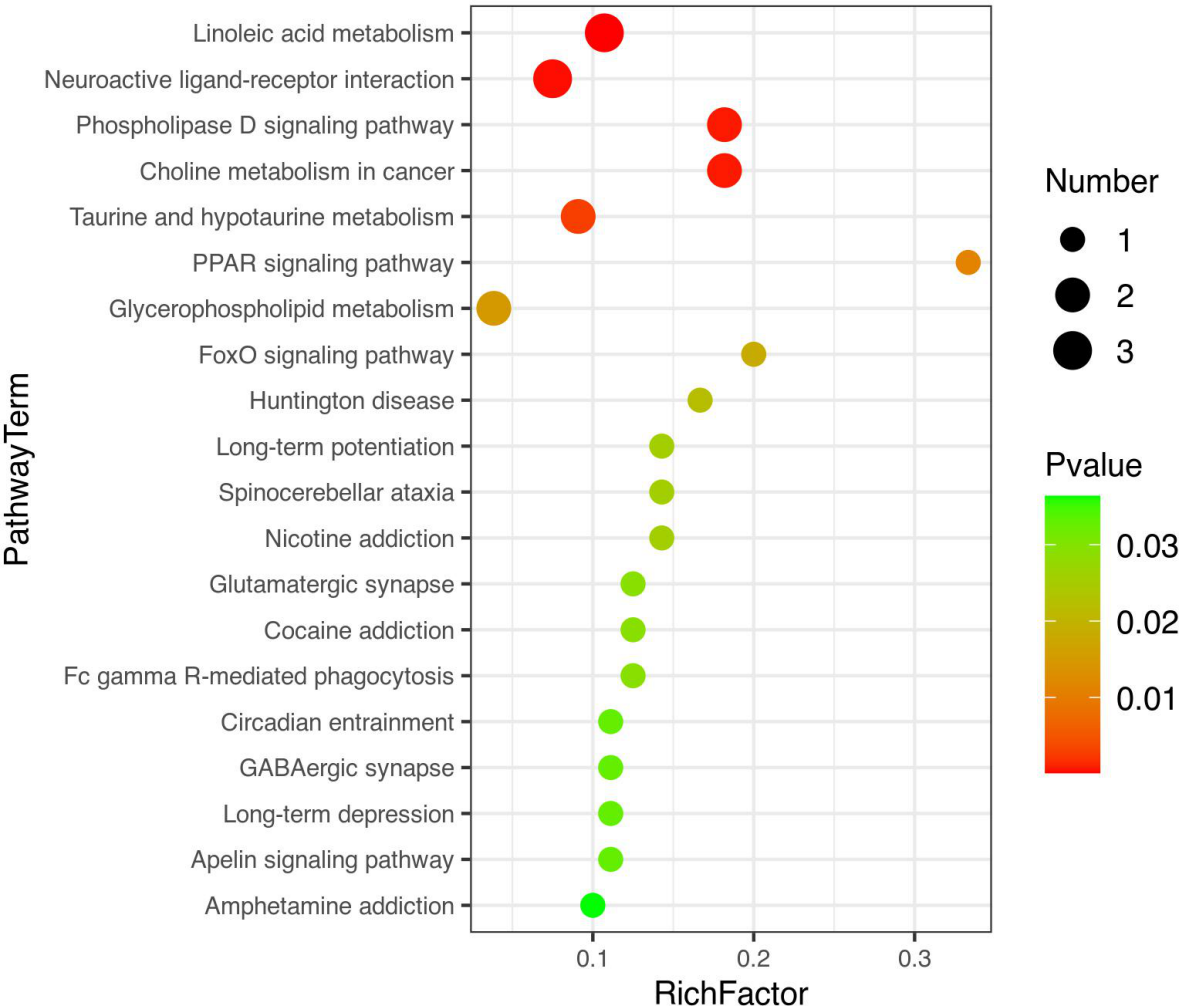
A bubble diagram of top-20 metabolic pathways. The color from green to red indicates that p-values decrease successively. The larger the point is, the more metabolites are enriched into the metabolic pathway.

**Figure 4** shows the PCA of all the samples, including the QCs. The QC samples were closely clustered in the middle of all the samples indicating that the analytical equipment used was stable and the experimental data was reliable. In the OPLS-DA, the intercepts of R2 and Q2 were 0.431 and -0.434, respectively, after 200 displacement tests. As shown in **Figure 5A**, the Q2 value was less than zero and there was no over-fitting of the model, which indicates that the model was reliable and effective. The OPLS-DA scores of the two groups showed a significant difference (**Figure 5B**). 103 significantly DEMs were found, of which 42 were up-regulated and 61 were down-regulated in the CPP group when compared to the normal group (>1-fold or <1-fold). All the 103 DEMs can be visualized *via* a volcano plot as shown in **Figure 6**. Metabolic pathway enrichment analysis of the DEMs was performed based on KEGG database. The top 20 metabolic pathways are shown in **Figure 7**.

### Bioinformatic analysis-integrated analysis of proteomics and metabolomics

**Figure 8:**
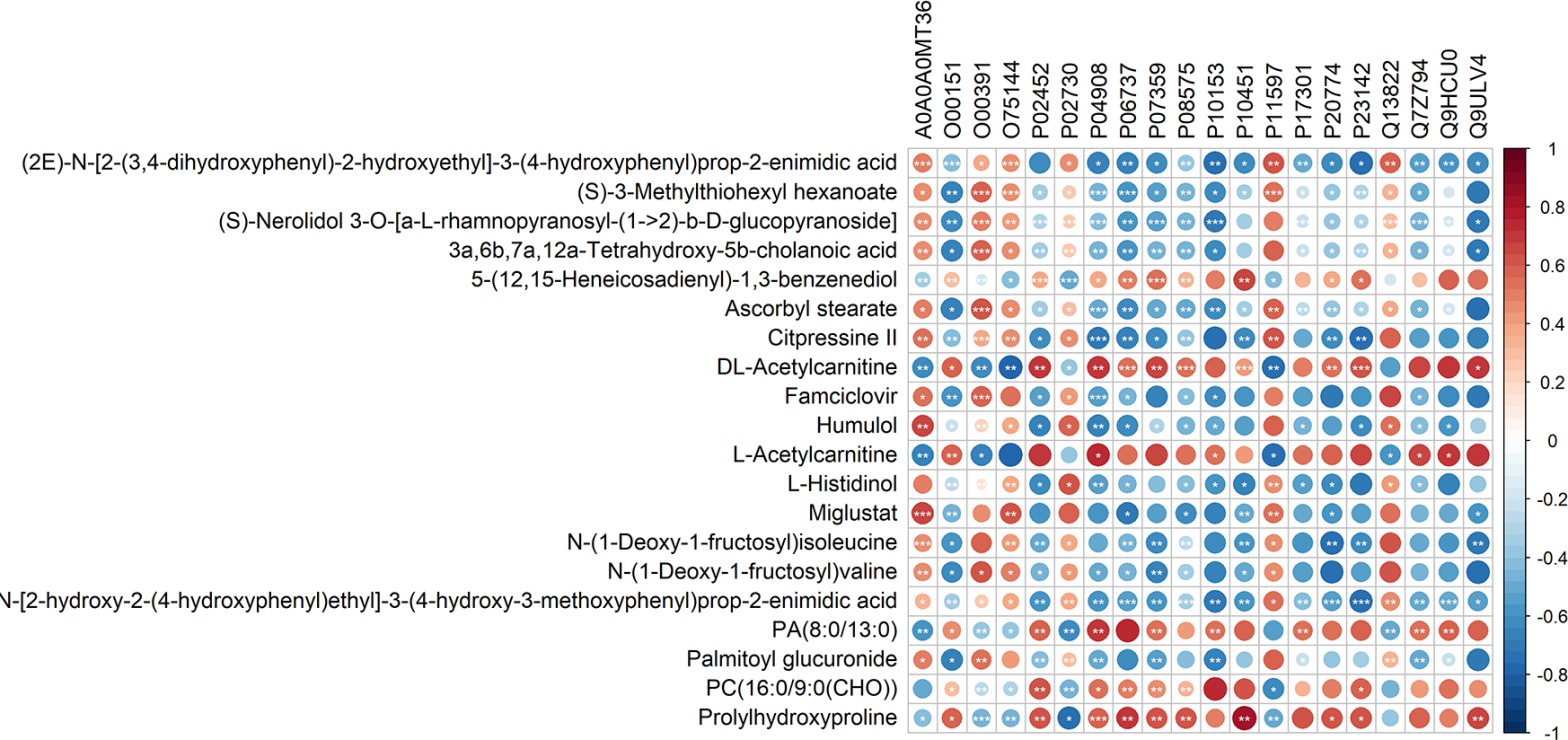
Correlation analysis of the differential proteins and metabolites. In the figure, red is positively correlated and blue is negatively correlated. *** represents correlation p value less than 0.001, ** represents correlation p value less than 0.01, and * represents correlation p value less than 0.05.

In our study, 10 girls with CPP and 10 age-matched healthy controls underwent simultaneous proteomic and metabolomic analysis, and the 10 pairs of data obtained were analyzed jointly. The top 20 relative content data of DEPs and DEMs were extracted based on the p-values, and the correlation between proteins and metabolites was calculated by Pearson correlation analysis and a correlation heatmap was drawn (**Figure 8**). The differential proteins and metabolites were simultaneously mapped to the KEGG database in order to obtain any common pathways and 6 were obtained (**Table 3**).

**Table 3:** Six pathways showing the differential proteins and metabolites involved.

Pathway	Pathway name	Gene	Metabolite
Hsa04931	Insulin resistance	PYGL	L-Acetylcarnitine DL-Acetylcarnitine
Hsa05231	Choline metabolism in cancer	RAC2	PC (16:1(9Z)/16:1(9Z)) LysoPC (17:0) Glycerophosphocholine
Hsa00270	Cysteine and methionine metabolism	GOT1	Phosphohydroxypyruvic acid
Hsa00400	Phenylalanine, tyrosine and tryptophan biosynthesis	GOT1	3α,6β,7α,12α-Tetrahydroxy-5β-cholanoic acid
Hsa00590	Arachidonic acid metabolism	GPX3	PC (16:1(9Z)/16:1(9Z))
Hsa00565	Ether lipid metabolism	ENPP2 PLA2G7	Glycerophosphocholine

**Figure 9:**
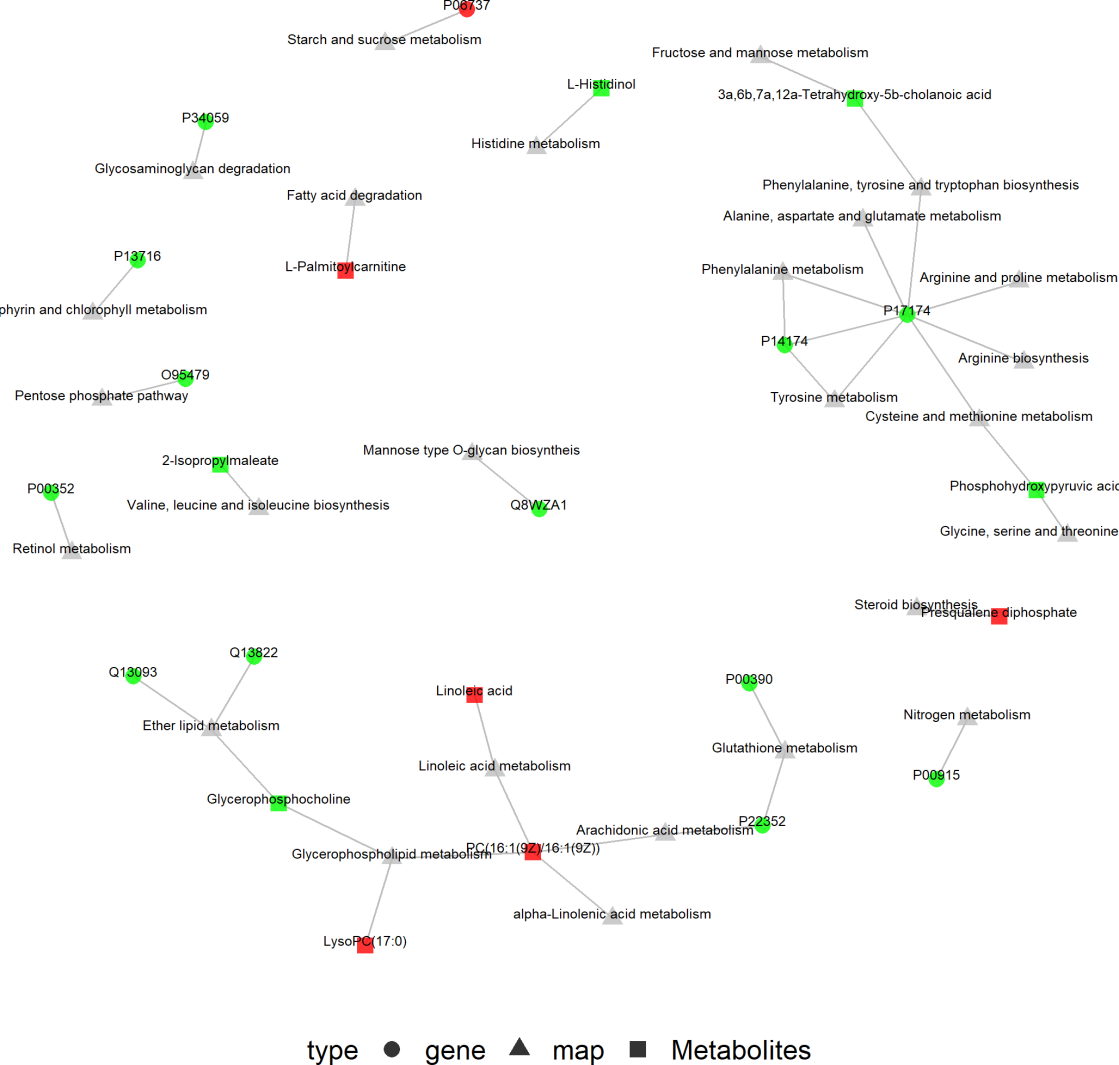
KGML network of the differential genes and metabolites. The squares represent genes and the triangles represent metabolites. Red represents up-regulated genes or metabolites, and bright green represents down-regulated genes or metabolites.

The KGML database is a sub-library of the KEGG database, which contains both the relationships of graph objects in the KEGG pathway and information regarding the lineal homologous genes in the KEGG genes database. Using this information, a network relationship between proteins and metabolites can be obtained, which is a convenient way to study the interactions between the proteome and metabolome in a systematic way, as shown in **Figure 9**. PC (16:1(9Z)/16:1(9Z)) was up-regulated in four of the metabolic pathways, including those involved in arachidonic acid, glycerophospholipid, linoleic acid and alpha-linolenic acid metabolism. The protein, P17174 (aspartate aminotransferase encoded by gene *GOT1*), was down-regulated in seven metabolic pathways involving the amino acids phenylalanine, tyrosine, tryptophan, arginine, alanine, aspartic and glutamic acids, cysteine, methionine, arginine and proline.

## Discussion

To the best of our knowledge, our study is the first to integrate proteomics and metabolomics to analyze serum samples obtained from girls with CPP. Using proteomic analysis, it was found that several different proteins in girls with CPP were mainly distributed in the extracellular region (48.5%) when compared with normal girls, suggesting that many of the active proteins played roles in these parts of the cells. MF analysis showed that the main functions associated with the differential proteins were related to their binding abilities. These results were consistent with a previous proteomic study of girls with CPP (24). The up-regulated proteins were mainly enriched in extracellular matrix organization, cell adhesion and cellular protein metabolic process, platelet degranulation and skeletal system development, which were related to pubertal growth and development. The down-regulated proteins were mainly enriched in immune response which might be involved in the development of CPP by participating in the regulation of sex hormones during puberty (25, 26).

In order to find the core proteins that are linked to CPP, the top 25 proteins with connectivity were selected to construct a PPI network and IGF-1 was found to have the highest connectivity. IGF-1 has been previously shown to have a predictive role as a biomarker in the diagnosis of girls with CPP (27). IGF2 and IGFBP1 were also thought to be involved in the process of puberty (28, 29). In addition, this study revealed other new candidate proteins such as MMP9, TIMP1, SPP1, CDC42, POSTN, COL1A1, COL6A1, COL2A1 and BMP1. Among them, MMP9, TIMP1, SPP1, CDC42, COL1A1, COL2A1, COL6A1 and BMP1 were previously correlated with the skeletal development and maturation (30–35). In addition, MMP9, TIMP1, SPP1 and POSTN were shown to play a role in regulating fat metabolism and insulin resistance (30, 36–38). MMP9, TIMP1, CDC42 and BMP1 were also involved in follicular development (39–43), suggesting that these proteins may be closely related to pubertal development. However, these proteins had not been previously reported to be related to CPP, and their mechanisms needs to be further studied.

Subsequently, a metabolomic analysis was performed and 103 DEMs were identified, which were mainly enriched in linoleic acid metabolism, neuroactive ligand receptor interaction, phospholipase D signaling pathway, choline metabolism in cancer, taurine and low taurine metabolism pathways. In the linoleic acid metabolic pathway, the serum levels of linoleic acid and its metabolic derivatives (9-HODE and 12,13-DHOME) in CPP girls were significantly higher than those found in the normal group. Lineolic acid is a polyunsaturated and is an essential fatty acid in humans where it stimulates insulin secretion (44). However, excessive long-term amounts of this fatty acid in the diet can aggravate a metabolic response resulting in an intestinal microflora imbalance in obese diabetic rats (45). Linoleic acid and its metabolic derivatives may be involved in CPP by regulating glucose and lipid metabolism.

In the neuroactive ligand receptor interaction pathway, sphingosine-1-phosphate (S1P), taurine and glutamate play important roles in neurotransmission and energy metabolism. The S1P/S1PR1/ceramide axis can activate the hunger signaling pathway in the hypothalamus through S1P so as to increase energy consumption and lipolysis, reduce food intake, thereby producing anti-obesity effects (46). Taurine is a sulfur amino acid that can induce browning of white adipose tissue, resulting in increased energy expenditure and adaptive thermogenesis (47). Taurine also has an anti-obesity effect through a combination of actions, including stimulating energy expenditure, improving lipid metabolism, suppressing appetite and inhibiting oxidative stress (48). In our study, the serum S1P and taurine levels of CPP girls were higher than those of the normal group, indicating that girls with CPP had enhanced energy metabolism and were in need extra energy. In addition, previous studies have shown that neuroactive ligand receptors in the neuroactive ligand receptor interaction pathway such as KISS1/KISS1R, TAC3/TACR3, NPY/NPYR and LEP/LEPR can play a role in the occurrence and development of CPP. Yang (49) conducted a metabonomic study using urine samples obtained from girls with CPP and found that several metabolites in their urine were correlated more with the activity of the nervous system rather than the endocrine system. A variety of neuroactive ligands and their receptors may act together in the CNS thereby promoting the occurrence and development of CPP. The neuroactive ligand receptor interaction may be an important metabolic pathway in CPP.

Finally, an integrated bioinformatic analysis of proteomics and metabolomics was carried out. KGML network analysis showed that PC (16:1(9Z)/16:1(9Z)) was involved in the metabolism of arachidonic acid, glycerophospholipid and linoleic and α-linolenic acids. PC (16:1(9Z)/16:1(9Z)) is a phosphatidylcholine and it acts on LysoPC (17:0) as well as arachidonic, linoleic and α-linolenic acids in four metabolic pathways. Excessive phosphatidylcholine would generate a large amount of arachidonic acid through the linoleic and arachidonic acid metabolic pathways. Increased arachidonic acid production would, in turn, generate excessive prostaglandins through the arachidonic acid metabolic pathways. Prostaglandins could further stimulate the release of LHRH, which would stimulate gonadotropin secretion and interact with sex hormones to affect follicular development and ovulation (50), thus playing an important role in the pathogenesis of CPP. Arachidonic acid, glycerophospholipid and linoleic acid metabolism may be important metabolic pathways involved in the development of puberty in girls. In addition, PC (16:1(9Z)/16:1(9Z)) may be a potentially important biomarker for CPP. However, there were some limitations to our study: 1. pubertal healthy girls were not included as controls in this study; 2. the sample size studied was small, and further a large sample of cohorts is needed to verify some of our findings; 3. the BMI, diet and other general living characteristics of the subjects were not controlled in this study, which might have caused some interference to the results obtained.

In conclusion, we conducted proteomics and metabolomics of serum samples of girls with CPP and found that they presented important differential proteins, metabolites and key metabolic pathways, which could provide novel ideas and clues to explore the pathogenesis of CPP. Subsequent PPI and KGML network analysis, yielded core proteins and metabolites that may be closely related to pubertal development and may be used as serum biomarkers for CPP. Arachidonic acid, glycerophospholipid and linoleic acid metabolism may also be additional metabolic pathways involved in the development of puberty in girls. However, more animal and cell experiments are needed to further clarify the role of these metabolic pathways in the pathogenesis of CPP.

## Data availability statement

Supplementary datasets to this article are available online - DOI for Figshare data: 10.6084/m9.figshare.20206184 (https://doi.org/10.6084/m9.figshare.20206184). The mass spectrometry proteomics data have been deposited to the ProteomeXchange Consortium (http://proteomecentral.proteomexchange.org) *via* the iProX partner repository (51) with the dataset identifier PXD035132.

## Ethics statement

The studies involving human participants were reviewed and approved by Scientific Ethics Committee of The First Affiliated Hospital of Guangxi Medical University in Nanning, China (2022 (KY-E-025)). Written informed consent to participate in this study was provided by the participants’ legal guardian/next of kin.

## Author contributions

DL and ML participated in study design and drafted the manuscript. ML and YC collected the cases and performed the laboratory assays. The final manuscript was approved by all the authors.

## Funding

The funds for this research were obtained from the First Affiliated Hospital of Guangxi Medical University Starting Fund for persons who are study-abroad returnees (Grant No. 2010001).

## Acknowledgments

The authors are grateful to all participants and thank Dr Dev Sooranna of Imperial College London for his guidance in English style of the manuscript.

## Conflict of interest

The authors declare that the research was conducted in the absence of any commercial or financial relationships that could be construed as a potential conflict of interest.

## Publisher’s note

All claims expressed in this article are solely those of the authors and do not necessarily represent those of their affiliated organizations, or those of the publisher, the editors and the reviewers. Any product that may be evaluated in this article, or claim that may be made by its manufacturer, is not guaranteed or endorsed by the publisher.

## Abbreviations

CPP, central precocious puberty; DEPs, differentially expressed proteins; DEMs, differentially expressed metabolites; HPGA, hypothalamic-pituitary-gonadal axis, EDCs, endocrine disrupting chemicals; COVID-19, coronavirus disease-19; KISS, kisspeptin; PVN, hypothalamic paraventricular nucleus; PCA, principal component analysis; OPLS-DA, orthogonal partial least-squares-discriminant analysis; VIP, variable importance in the projection; LH, luteinizing hormone; FSH, follicle-stimulating hormone; GnRH, gonadotropin-releasing hormone; DIA, data-independent acquisition; CNS, central nervous system; GO, Gene Ontology; KEGG, Kyoto Encyclopedia of Genes and Genomes; FDR, false discovery rate; BP, biological process; CC, cellular component; MF, molecular function; PPI, protein-protein interaction; S1P, sphingosine-1-phosphate.
